# High Added Sugars Intake among US Adults: Characteristics, Eating Occasions, and Top Sources, 2015–2018

**DOI:** 10.3390/nu15020265

**Published:** 2023-01-04

**Authors:** Seung Hee Lee, Lixia Zhao, Sohyun Park, Latetia V. Moore, Heather C. Hamner, Deborah A. Galuska, Heidi M. Blanck

**Affiliations:** Division of Nutrition, Physical Activity, and Obesity (DNPAO), National Center for Chronic Disease Prevention and Health Promotion (NCCDPHP), Centers for Disease Control and Prevention (CDC), 4770 Buford Highway, NE, Atlanta, GA 30341, USA

**Keywords:** added sugars, adults, dietary intake, National Health and Nutrition Examination Survey

## Abstract

The 2020–2025 Dietary Guidelines for Americans (DGA) recommends less than 10% of total daily calories come from added sugars. However, many adults overconsume added sugars putting them at risk for poor health outcomes. We examined characteristics of high added sugars consumers among US adults (≥20 years) and described their top 10 sources of added sugars intake using National Health and Nutrition Examination Survey 2015–2018 data (*n* = 9647). We defined high consumers as consuming >15% of daily calories from added sugars (1.5 times higher than the DGA). We used the National Cancer Institute method to estimate usual intake of energy and percent of calories from added sugars. Top 10 sources were identified based on their percentage contribution to total added sugars intake on a given day. T-tests were used to examine differences by age, sex, race/ethnicity, education, income, marital status, and weight status. Overall, mean usual total energy intake and added sugars intake was 2068 kcal/day and 264 kcal/day, respectively, and 30% of adults were classified as high consumers. The prevalence of high added sugars consumers was significantly higher among 20–30-year-olds (29%), 31–50-year-olds (33%), and 51–70-year-olds (29%) than those aged ≥70 years (22%); non-Hispanic Black (39%) and non-Hispanic White (31%) adults than Hispanics (26%); adults with <high school (37%), high school/GED (38%), or some college (34%) than adults with college or higher (15%); adults living in lower-income households (39% for federal poverty income ratio < 130% and 35% for 130%–<350%) than high-income households (21%). The prevalence of high consumers did not differ by sex or weight status. Top sources of added sugars were sweetened beverages (42%), tea (12%), sweet bakery products (11%), and jams/syrups/sugars (7%). Our findings can inform intervention efforts to decrease added sugars intake to support health.

## 1. Introduction 

Added sugars within foods and beverages provide excess calories with little nutritional value and increase the risk of chronic diseases including obesity [1], hypertension [2], and dyslipidemia [3], and cardiovascular disease mortality [4]. Added sugars include sugars that are added during the processing of foods, foods packaged as sweeteners (e.g., table sugar), sugars from syrups and honey, and sugars from concentrated fruit or vegetable juices [5]. The 2020–2025 Dietary Guidelines for Americans (DGA) state that added sugars should account for less than 10% of total daily calories [6]. Based on 2015–2016 National Health and Nutrition Examination Survey (NHANES), 13% of total daily calories were from added sugars among US adults [7], which declined from 18% in NHANES 1999–2000 [8].

A previous analysis found that added sugars intake among US adults is high. In 2017–2018, mean added sugars intake among US adults aged 20 years and older was 17.1 teaspoons (tsp)/d, 19.2 tsp for men and 15.1 tsp for women [9], which were much higher than the American Heart Association’s recommendation to consume less than 9 tsp and 6 tsp for men and women, respectively [10]. Among adults who exceed the DGA recommendation for limiting added sugars (i.e., consuming ≥10% of total calories from added sugars), 19.4% of calories were from added sugars in 2016–2017 [11]. In addition, Park et al. found that those who consume high amounts of added sugars (men: ≥22.0 tsp/d; women ≥ 14.6 tsp/d) had higher odds of being younger, less educated, and had lower income than those with lower consumption [12].

To address the high consumption of added sugars among Americans, one of the US Department of Health and Human Services’ Healthy People 2030 Objectives is to “Reduce consumption of added sugars by people aged 2 years and over. [13]” Although some studies have shown added sugars consumption patterns and its sources among all US adults [11], little information is available on the characteristics of high added sugars consumers among US adults, who might most benefit from interventions to reduce added sugars because of increased health risk associated with higher consumption [11]. Therefore, we examined characteristics of high added sugars consumers (i.e., >15% of their calories from added sugars, 1.5 times higher than the DGA recommendation) among a nationally representative sample of US adults (≥20 years) and described the eating occasions and the top 10 sources of added sugars intake.

## 2. Methods

### 2.1. Data Source and Participants

This study used data from NHANES 2015–2018. NHANES is a cross-sectional, nationally representative, multistage survey of the civilian, noninstitutionalized persons in the United States [14]. The survey combines interviews and an in-person health examination with a 24 h (24-h) dietary recall conducted in a mobile examination center, and a second 24 h dietary recall collected by telephone 3–10 days later. Dietary intake was self-reported. Among participants aged ≥20 years who had a reliable in person 24 h (Day 1) dietary recall (*n* = 9759), we excluded those with missing data on added sugars intake (*n* = 1) and those who were pregnant (*n* = 111), leaving an analytic sample of 9647 adults.

### 2.2. Outcome Variables

The United States Department of Agriculture (USDA)’s Food Patterns Equivalents Database (FPED) for 2015–2016, and 2017–2018 were used to disaggregate all foods and beverages into their components and quantify added sugars consumption. The total daily amount of added sugars intake for each participant, in tsp equivalents, were summed across all foods and beverages that were reported. We converted tsp equivalents into grams by multiplying by 4.2 g (g)/tsp equivalent, then calculated calories from added sugars by multiplying 4 kcal/g. To obtain the percentage of calories (% kcal) from added sugars, we divided the calories from added sugars by the total energy intake (in kcal/d), which was provided in NHANES dietary data files. The calculations were done separately for the first and second 24 h dietary recall. We defined high added sugars consumers as those who consumed >15% kcal from added sugars, which is 1.5 times higher than the DGA recommendation (i.e., <10% of total daily calorie intake).

We used the National Cancer Institute (NCI) [15] method to estimate usual mean total energy intake and proportion of participants who consumed >15% kcal from added sugars (hereafter, referred to as high consumers). Because nearly every participant consumed added sugars and energy daily, we employed the amount only model accounting for weekend vs. weekday effects. The NCI method requires that at least some respondents have two days of reported nutrient intake to estimate between-and within-person variations [15]. In our study, 8247 (86.7%) participants provided two reliable 24 h dietary recalls. The models for estimating the distribution of usual intake were adjusted for an indicator of sequence number (first- vs. second-day of dietary recall); and day of the week the recall was collected (weekday vs. weekends [Friday–Sunday]).

### 2.3. Explanatory Variables

The explanatory variables included age (20–30 years, 31–50 years, 51–70 years, ≥71 years), sex (female, male), race/ethnicity groups with sufficient sample size to calculate reliable estimates (non-Hispanic [NH] Black, Hispanic, NH Other, NH White), education (less than high school, high school graduate/General Education Development, some college, college graduate or higher), marital status (not married, married/domestic partnership), federal poverty income ratio (PIR) (<130%, 130% to 350%, ≥350%), which is the ratio of family income to poverty level, which was calculated by dividing family income by the poverty level guidelines specific to the survey year [16] Weight status was categorized as underweight/healthy weight (Body Mass Index [BMI] < 25 kg/m^2^); overweight (BMI 25 to <30 kg/m^2^); obesity (BMI ≥ 30 kg/m^2^); and severe obesity (BMI ≥ 40 kg/m^2^) [17] Severe obesity is a subset of the obesity category. About 1.4% of adults were classified as underweight, thus due to the small sample size, we combined underweight with healthy weight.

To examine eating occasions related to added sugars and sources of added sugars among high consumers, we used the Day 1 24 h recall to identify high consumers (*n* = 3236). For each food and beverage reported, a list of 20 different names of eating occasions was provided to the respondent for selection. We categorized those eating occasions as four categories: (1) breakfast (i.e., breakfast, desayuno, and almuerzo); (2) lunch (i.e., lunch, comida, and brunch); (3) dinner (i.e., dinner, supper, and cena); and (4) snack (i.e., snack, merienda, entre comida, botana, bocadillo, tentempie, extended consumption, drink, and bebida). For each eating occasion, the amounts of added sugars intake (in kcal) for each participant on Day 1 dietary recall were summed across all foods and beverages reported at the eating occasion. The percentage (%) contribution of added sugars at each eating occasion was calculated as the sum of the amount of added sugars consumed at the eating occasion for all participants, divided by the sum of added sugars consumed from all foods and beverages for all participants, and multiplied by 100.

To identify top food sources/groups contributing to added sugars consumption, USDA What We Eat In America (WWEIA) food categories for each NHANES cycle were collapsed into 23 categories (15 food, 7 beverage, and 1 other) (Appendix A). Food groups were ranked based on their percentage contribution to total added sugars intake on Day 1 dietary data, calculated as the sum of added sugars consumed from a specific food group, divided by the sum of added sugars consumed from all food groups, and multiplied by 100.

### 2.4. Statistical Analyses

Descriptive analyses were presented as weighted means and standard errors (SE) or weighted percentage and SE for sociodemographic characteristics and weight status. T-tests were used to compare mean differences of usual total energy intake or % calories from added sugars between groups (e.g., male vs. female, NH White vs. Hispanic). All tests were two sided, and *p*-values < 0.05 were considered statistically significant. All statistical analyses were performed in SAS 9.4 (SAS Institute Inc., Cary, NC, USA) or SAS-callable SUDAAN (RTI international, Research Triangle Park, NC, USA) using combined dietary sample weights from two survey cycles to account for complex sampling design and non-response. Balanced repeated replication was used to calculate standard errors and 95% confidence intervals (CIs). Sensitivity analyses were conducted for the eating occasions and top sources of added sugars analyses to detect any meaningful differences by age, sex, and race/ethnicity.

## 3. Results

Of the 9647 adults, 34% were 51–70 years old, about half (51%) were female, 63% were NH White, 31% had college or higher education, 63% were married or in domestic partnership, 43% had PIR ≥ 350%, and 41% had obesity (Table 1). US adults consumed an average of 2068 kcal/day during 2015–2018 with a usual mean total energy intake from added sugars of 264 kcal/day (Table 1).

In 2015–2018, 29.9% of adults were classified as high consumers (i.e., >15% of total calories from added sugars) (Table 2). The prevalence of high consumers was significantly higher among 20–30-year-olds (29%), 31–50-year-olds (33%), and 51–70-year-olds (29%) than those aged ≥71 years (22%); non-Hispanic Black (39%) and non-Hispanic White (31%) adults than Hispanics (26%); adults with <high school (37%), high school/GED (38%), or some college (34%) than adults with college or higher (15%); not married adults (33%) than adults who were married or in domestic partnership (28%); adults living in lower-income households (39% for PIR < 130% and 35% for PIR 130%–<350%) than high-income households (21%). The prevalence of high consumers did not differ by sex or weight status (Table 2).

By eating occasions among high consumers of added sugars, the contribution of energy intake from added sugars was the highest during snacking (225 kcal, 43%), followed by dinner (124 kcal, 24%), lunch (98 kcal, 19%), and breakfast (79 kcal, 15%) on a given day (Table 3). Of the top 10 sources of added sugars among the high consumers, over half of the contribution came from beverages: 42% from sweetened beverages and 12% from tea, followed by sweet bakery products (11%), jams/syrups/sugars (7%), candy (5%), other desserts (4%), alcoholic beverages (3%), mixed dishes (3%), ready-to-eat-cereals (2%), and fats and oils (2%) (Table 4). In sensitivity analyses, there were no differences in added sugars intake patterns for eating occasions or top food sources when stratified by age, sex, and race/ethnicity groups among high consumers (data not shown).

## 4. Discussion

In 2015–2018, about 3 in 10 US adults aged ≥20 years were high consumers who consumed more than 15% of total daily calories from added sugars. Moreover, the prevalence of high added sugars consumers differed by age, race/ethnicity, education, marital status and income status. For example, we found that the prevalence of high added sugars consumers was significantly higher among younger adults (vs. adults aged ≥71 years), adults with lower income (vs. PIR ≥ 350%), adults with lower education (vs. college or higher), and NH White or NH Black adults (vs. Hispanics) in 2015–2018. Our findings are consistent with previous findings that showed the intake of added sugars was inversely related to age, educational status, and family income [12,18], however, our finding that NH Black and NH White adults had a higher prevalence of high added sugars consumers compared to Hispanic counterparts is inconsistent with some other studies [9,12]. The differences observed may be due to our focus on high consumers vs. all US adults [8], the temporality (2015–2018 vs. 2010) [10], and different methods of data collection between NHANES’ 24 h recall compared to NHIS’ 26-item Dietary Screening Questionnaire [10]. In addition, similar to our results showing no differences in the prevalence of high consumers between men and women, a previous study also found no difference in meeting the DGA recommendation for limiting added sugars by sex [11].

By eating occasions, we found that mean calorie intake of added sugars was highest during snacking among high added sugars consumers, which contributed 43% of calories from added sugars (225 kcal). This is approximately equivalent to a 16 oz bottle of soda or 2 × 2 inches of brownie (i.e., 48 g) [19]. This pattern is similar to findings from other countries, such as Australia [20] and Canada [21]. Based on 2009–2012 NHANES, adults aged 19–59 years consumed snacks 2.3 times a day (493 kcal/day) and adults aged ≥60 years consumed snacks 2.1 times a day (347 kcal/day) [22] The majority of energy intake from snacks was from sources generally considered less healthy, such as, desserts and sweets, salty snacks, and sugar-sweetened beverages (SSBs). Previous study had shown nearly one-third of eating occasions occurred at non-designated eating places, such as a couch in front of a television or a workspace [23]. In another study, snacking was more likely at work than at home, however, more of the calories from added sugars were consumed at home rather than away from home [24]. While we were not able to identify whether different type of snacks are consumed at specific locations, understanding whether different types of snacks are consumed at different types of eating occasions and why may help with designing tailored messaging and intervention strategies to reduce added sugars consumption.

In this study, the top two sources of added sugars were SSBs—sweetened beverages (42%) and tea (12%). A previous report from 2015–2016 NHANES found the same top sources; among adults who exceeded the DGA recommendation for limiting added sugars (i.e., ≥10% calories from added sugars), men consumed a total of 125 g of added sugars and about 52 g and 15 g of those added sugars were consumed from sweetened beverages and tea, respectively; in comparison, women consumed a total of 88 g of added sugars, and about 27 g and 11 g of added sugars from sweetened beverages and tea, respectively [11]. Investigating the top sources of added sugars, particularly among high consumers, may help identify specific foods or beverages to target in intervention efforts. A tailored approach can be layered with existing population-based nutrition standards that are in place in many workplaces and communities [25]. Product reformulation could reduce the amount of added sugars in sweetened beverages, packaged tea products (e.g., canned or bottled tea), and sweet bakery products. In addition, educating individuals to consider consuming less food and beverages with added sugars (e.g., smaller packages or cup sizes) may be approaches in reducing the total added sugars intake among high consumers.

This study has strengths and weaknesses. A strength of this study is the use of the NCI methods for calculating usual intake to identify the prevalence of high consumers of added sugars. By using two 24 h dietary recalls, estimates reflect usual added sugars intake among US adults after considering individual variances. Another strength is the use of a large, nationally representative sample of US adults. A limitation of this study is that we used data from one 24 h dietary recall to identify eating occasions and top sources of added sugars among high consumers, which may not represent the usual consumption pattern among high consumers due to day-to-day variation in dietary intake. In addition, dietary intake from recalls may underestimate or overestimate the actual intake.

In conclusion, approximately 3 in 10 US adults were classified as high consumers of added sugars in 2015–2018, meaning that 15% or more of their daily caloric intake came from added sugars, which offer little to no nutritional value. These findings can inform future health communication and interventions strategies to reduce added sugars consumption by identifying specific populations, eating occasions, and food/beverage sources that might merit additional focus.

## Figures and Tables

**Table 1:** Sociodemographic characteristics, usual mean total energy intake, and usual mean energy intake from added sugars among US adults aged ≥20 years, NHANES 2015–2018.

Characteristics	*n* ^a^	% ^b^	Usual Mean Total Energy Intake		Usual Mean Energy from Added Sugars Intake	
			kcal/Day	95% CI	kcal/Day	95% CI
Total	9647	100	2068	2045, 2092	264	255, 274
Age						
20–30 years	1623	20.1	2143 *	2092, 2193	269 *	250, 287
31–50 years	3067	33.2	2151 *	2107, 2196	289 *	273, 305
51–70 years	3445	33.9	2036 *	1999, 2074	257 *	243, 272
≥71 years (Reference)	1512	12.7	1816	1767, 1866	212	202, 221
Sex						
Male	4722	48.6	2392 *	2359, 2424	302 *	288, 316
Female (Reference)	4925	51.4	1765	1733, 1797	230	219, 241
Race/ethnicity						
Black, non-Hispanic	2161	11.2	1992 *	1947, 2037	286 *	272, 300
Hispanic (Reference)	2567	15.3	2083	2036, 2129	251	238, 263
Other, non-Hispanic	1544	10.0	2030	1973, 2087	221 *	201, 241
White, non-Hispanic	3375	63.4	2085	2051, 2119	271 *	258, 283
Education (*n* = 9637)						
<High school	2018	12.2	2005 *	1935, 2074	290 *	268, 313
High school/GED	2231	24.6	2030 *	1982, 2078	291 *	276, 306
Some college	3009	32.1	2097	2049, 2146	285 *	269, 300
College or higher (Reference)	2379	31.1	2097	2054, 2141	215	204, 226
Marital Status (*n* = 9642)						
Married/Domestic partnership (Reference)	5747	62.5	2085	2057, 2114	257	245, 268
Not married	3895	37.5	2037	1998, 2076	277 *	263, 290
Federal poverty income ratio ^c^ (*n* = 8622)						
<130%	2567	20.8	1990 *	1939, 2042	290 *	274, 306
130%–<350%	3513	36.1	2091	2043, 2140	288 *	276, 300
≥350% (Reference)	2542	43.1	2109	2065, 2153	236	224, 248
Weight status ^d^ (*n* = 9549)						
Underweight /healthy weight (Reference)	2496	26.8	2070	2020, 2121	264	250, 278
Overweight	3064	31.8	2087	2042, 2133	260	243, 277
Obesity	3989	41.4	2057	2023, 2090	270	259, 280
Severe obesity ^e^	834	8.2	2077	1974, 2179	293	272, 314

Abbreviation: CI, confidence interval; GED, General Education Development. * Significantly differ from the reference group (*p* < 0.05). ^a^ Unweighted sample size. ^b^ Weighted percent may not add up to 100% because of rounding. ^c^ Federal poverty income ratio, is the ratio of family income to poverty, which was calculated by dividing family income by the poverty guidelines specific to the survey year. ^d^ Weight status was based on calculated body mass index (BMI) (kg/m^2^) from measured weight and height data: underweight/healthy weight (BMI < 25 kg/m^2^); overweight (BMI 25 to <30 kg/m^2^); obesity (BMI ≥ 30 kg/m^2^); and severe obesity (BMI ≥ 40 kg/m^2^). ^e^ Severe obesity category is a subset of obesity category.

**Table 2:** Characteristics of US adults aged ≥20 years consuming greater than 15 % energy from added sugars, NHANES 2015–2018, usual intake.

Characteristics	Consuming >15 % Energy from Added Sugars		
	Prevalence (%)	95% CI	
Total (*n* = 9647)	29.9	27.5, 32.3	
Age			
20–30 years	29.2 *	24.1, 34.3	
31–50 years	33.0 *	30.3, 35.8	
51–70 years	29.3 *	25.5, 33.1	
≥71 years (Reference)	22.4	19.0, 25.7	
Sex			
Male (Reference)	29.2	26.5, 31.9	
Female	30.7	27.4, 34.1	
Race/ethnicity			
Black, non-Hispanic	39.1 *	35.3, 42.8	
Hispanic (Reference)	25.9	22.6, 29.2	
Other, non-Hispanic	19.4 *	15.0, 23.8	
White, non-Hispanic	30.8 *	28.0, 33.6	
Education (*n* = 9637)			
<High school	36.8 *	32.4, 41.2	
High school/GED	38.3 *	34.8, 41.7	
Some college	34.0 *	31.2, 36.7	
College or higher (Reference)	15.0	12.3, 17.6	
Marital Status (*n* = 9642)			
Married/Domestic partnership (Reference)	27.8	25.0, 30.5	
Not married	33.4 *	30.1, 36.8	
Federal poverty income ratio ^a^ (*n* = 8622)			
<130%	38.8 *	35.0, 42.6	
130%–<350%	35.1 *	32.4, 37.9	
≥350% (Reference)	20.9	17.9, 23.9	
Weight status ^b^ (*n* = 9549)			
Underweight /healthy weight (Reference)	30.1	26.8, 33.4	
Overweight	28.2	24.3, 32.2	
Obesity	31.6	28.8, 34.4	
Severe obesity ^c^	36.9	31.8, 42.0	

Abbreviation: CI, confidence interval; GED, General Education Development. * Significantly differ from the reference group (*p* < 0.05). ^a^ Federal poverty income ratio, is the ratio of family income to poverty level, which was calculated by dividing family income by the poverty guidelines specific to the survey year. ^b^ Weight status was based on calculated body mass index (BMI) (kg/m^2^) from measured weight and height data: underweight/healthy weight (BMI < 25 kg/m^2^); overweight (BMI 25 to <30 kg/m^2^); obesity (BMI ≥ 30 kg/m^2^); and severe obesity (BMI ≥ 40 kg/m^2^). ^c^ Severe obesity category is a subset of obesity category: it is not independent from the obesity category and thus cannot be compared to this group.

**Table 3:** Mean energy from added sugars intake by eating occasions among high consumers ^a^, NHANES 2015–2018, Day 1 dietary recall (*n* = 3236) ^b^.

	Among High Added Sugars Consumers	
Eating Occasion	Mean (kcal/d) ± SE	% Contribution ^c^ ± SE
Snack	225 ± 8.2	42.8 ± 1.2
Dinner	124 ± 4.9	23.5 ± 0.8
Lunch	98 ± 3.9	18.7 ± 0.8
Breakfast	79 ± 3.3	15.0 ± 0.5

Abbreviation: SE, standard error. ^a^ High added sugars consumers were defined as those who consumed greater than 15% of total calories from added sugars, which is 1.5 times higher than DGA recommendation (i.e., <10% of total daily calorie intake). ^b^ To examine eating occasions related to added sugars, we used the Day 1 24 h recall to identify high consumers. ^c^ The percentage (%) of added sugars consumed was calculated as the sum of the amount of added sugars from the foods and beverages consumed at each eating occasion for all person in the designated group, divided by the sum of added sugars consumed from all foods and beverages for all person in the designated group multiplied by 100.

**Table 4:** Top 10 sources of added sugars and mean calorie intake from top sources among high consumers of added sugars ^a^ among US adults, overall and by race/ethnicity, NHANES 2015–2018, Day 1 dietary recall (*n* = 3236) ^b^.

Ranking	Food Category	% of Added Sugars ± SE	Mean (kcal) ± SE
1	Sweetened Beverages ^c^	41.7 ± 1.2	219.5 ± 8.2
2	Tea	11.5 ±1.0	60.3 ± 5.4
3	Sweet Bakery Products	10.8 ± 0.6	56.8 ± 3.3
4	Jams/Syrups/Sugars	7.1 ± 0.5	37.5 ± 2.8
5	Candy	5.3 ± 0.4	27.8 ± 2.2
6	Other Desserts	4.1 ± 0.3	21.6 ± 1.6
7	Alcoholic Beverages	2.8 ± 0.6	14.8 ± 2.9
8	Mixed Dishes	2.7 ± 0.1	14.0 ± 0.6
9	Ready-to-eat-cereals	2.0 ± 0.2	10.7 ± 0.9
10	Fats and Oils	2.0 ± 0.3	10.5 ± 1.4

Abbreviation: SE, standard error. ^a^ High added sugars consumers were defined as those who consumed greater than 15% of total calories from added sugars, which is 1.5 times higher than DGA recommendation (i.e., <10% of total daily calorie intake). ^b^ To examine top sources of added sugars we used the Day 1 24 h recall to identify high consumers. ^c^ Includes soft drinks, fruit drinks, sport and energy drinks, nutritional beverages, smoothies and grain drinks.

## Data Availability

The data are publicly available online (https://wwwn.cdc.gov/nchs/nhanes/Default.aspx; accessed on 28 December 2022).

## Appendix A

**Table A1:** Breakdown of food groups that contribute to intake of added sugars determined by What We Eat in America (WWEIA).

Food Group	Food Types in Food Groups
Bread, Rolls, Tortillas	Yeast breads, rolls and buns, bagels and English muffins, tortillas
Candy	Candy containing chocolate, candy not containing chocolate
Condiments and Sauces	Tomato-based condiments, soy-based condiments, mustard and other condiments, olives, pickles, pickled vegetables, pasta sauces, tomato-based, dips, gravies, other sauces
Cooked Grains and Cereals	Rice, pasta, noodles, cooked grains, oatmeal, grits and other cooked cereals
Cured Meats/Poultry	Cold cuts and cured meats, bacon, frankfurters, sausages
Fats and Oils	Butter and animal fats, margarine, cream cheese, sour cream, whipped cream, cream and cream substitutes, mayonnaise Salad dressings and vegetable oils
Jams/Syrups/Sugars	Sugars, honey, sugar substitutes, jams, syrups, toppings
Mixed Dishes	Mixed dishes and pizza, all varieties, mixed dishes-sandwiches, all varieties
Other Desserts	Ice cream and frozen dairy desserts, pudding, gelatins, ices, sorbets
Protein Foods	Cheese, meats, poultry, seafood, eggs, plant-based protein foods
Quick Breads and Bread Products	Biscuits, muffins, quick breads, pancakes, waffles, French toast
Ready-to-eat-cereals	Ready-to-eat cereal, higher sugar (>21.2 g/100g), Ready-to-eat cereal, lower sugar (≤21.2 g/100g)
Snack/Meal Bars and crackers	Cereal bars, nutrition bars, crackers
Sweet Bakery Products	Cakes, pies, cookies, brownies, doughnuts, sweet rolls, pastries
Yogurt	Yogurt regular, Greek
**Beverage Group**	
Alcoholic beverages	Beer, wine, liquor, and cocktails
Coffee	Coffee ^a^
Dairy Drinks and Substitutes	Milk shakes and other dairy drinks, milk substitutes
Flavored milk	Flavored milk-whole, reduced fat, low fat, nonfat
Flavored and enhanced water	Flavored or carbonated water, enhanced or fortified water
Sweetened Beverages	Soft drinks, fruit drinks, sport and energy drinks, nutritional beverages, smoothies, and grain drinks
Tea	Tea ^b^
**Other**	Fruit, vegetables, 100% juice, diet beverages, plain water, baby foods and formulas, other

^a^ Examples of coffee include instant coffee with or without caffeine, sweetened with sugar/low calorie sweetener, coffee drinks such as cappuccino, iced latte, mocha with or without whipped cream, and frozen coffee drinks. ^b^ Examples of tea include Iced tea with lemonade, bottled green/black tea, brewed green/black/herbal tea sweetened with sugar, Chai tea with milk/sweetener, and various instant teas with or without caffeine, sweetened or unsweetened.
